# Adherence to a healthy diet in relation to cardiovascular incidence and risk markers: evidence from the Caerphilly Prospective Study

**DOI:** 10.1007/s00394-017-1408-0

**Published:** 2017-03-14

**Authors:** Elly Mertens, Oonagh Markey, Johanna M. Geleijnse, Julie A. Lovegrove, D. Ian Givens

**Affiliations:** 1 Division of Human Nutrition, Wageningen University, P.O. Box 8129, 6700 EV Wageningen, The Netherlands; 2 Hugh Sinclair Unit of Human Nutrition, Department of Food and Nutritional Sciences and Institute for Cardiovascular and Metabolic Research (ICMR), University of Reading, Reading, RG6 6AP UK; 3 School of Sport, Exercise and Health Sciences, Loughborough University, Loughborough, LE11 3TU UK; 4 Food Production and Quality Division, School of Agriculture, Policy and Development, Faculty of Life Sciences, University of Reading, Reading, RG6 6AP UK

**Keywords:** Dietary adherence, Dietary approaches to stop hypertension score, Alternative healthy eating index-2010, Cardiovascular disease, Aortic pulse wave velocity

## Abstract

**Purpose:**

Epidemiological findings indicate that higher adherence to a healthy diet may lower cardiovascular disease (CVD) risk. The present study aimed to investigate whether adherence to a healthy diet, assessed by the Healthy Diet Indicator (HDI), Dietary Approaches to Stop Hypertension (DASH) score, and Alternative Healthy Eating Index 2010 (AHEI-2010), was associated with CVD incidence and risk markers.

**Methods:**

Included in the present analyses were data from 1867 middle-aged men, aged 56.7 ± 4.5 years at baseline, recruited into the Caerphilly Prospective Study. Adherence to a healthy diet was examined in relation to CVD, coronary heart disease (CHD), and stroke incidence (Cox regression), and risk markers (linear regression) with adjustment for relevant confounders.

**Results:**

The DASH score was inversely associated with CVD [hazard ratio (HR) 0.81; 95% confidence interval (CI) 0.66, 0.99], and stroke (HR 0.61; 95% CI 0.42, 0.88) incidence, but not with CHD after an average of 16.6 year follow-up, and with diastolic blood pressure, after 12 year follow-up. The AHEI-2010 was inversely associated with stroke (HR 0.66; 95% CI 0.42, 0.88) incidence, aortic pulse wave velocity, and C-reactive protein. The HDI was not associated with any single outcome.

**Conclusions:**

Higher DASH and AHEI-2010 scores were associated with lower CVD and stroke risk, and favourable cardiovascular health outcomes, suggesting that encouraging middle-aged men to comply with the dietary recommendations for a healthy diet may have important implications for future vascular disease and population health.

**Electronic supplementary material:**

The online version of this article (doi:10.1007/s00394-017-1408-0) contains supplementary material, which is available to authorized users.

## Introduction

Diet has a fundamental role to play in the development and prevention of cardiovascular disease (CVD), which is the leading contributor to mortality worldwide [1]. While traditional epidemiological research has largely focused on single nutrients or foods, the more recent studies of dietary patterns have allowed for reflection on both the complexity and the synergies of food and nutrient intake [2, 3]. It has been documented that an overall healthy dietary pattern will be more predictive of disease risk than a single nutrient or food group [4]. Meta-analysis based on 15 longitudinal cohort studies established that higher adherence to a healthy diet was associated with a significant CVD risk reduction in a general population, showing a pooled risk ratio of 0.78 (95% confidence interval 0.75 and 0.81) [5]. A diet score is a practical application to assess dietary patterns in terms of the degree of adherence to the dietary recommendations for a healthy diet [6]. In recent years, numerous diet scores aimed at assessing diet quality have emerged; however, varying approaches in the scoring and components included are used due to the ambiguous definition of a healthy diet [7]. The Healthy Diet Indicator (HDI) is an instrument to measure adherence to the dietary guidelines of the World Health Organisation (WHO) [8], and has previously been used to study the relationship with CVD [9, 10]. The Dietary Approaches to Stop Hypertension (DASH) is a recognised dietary recommendation targeting blood pressure (BP) [11], and higher adherence was associated with a lower CVD risk [9]. Clinical trials have also demonstrated that adherence to the DASH diet has a beneficial influence on CVD risk markers [12]. The Alternative Healthy Eating Index-2010 (AHEI-2010) includes all food and nutrients that are the most predictive of chronic disease [13]. Higher scores were associated with CVD risk reductions in US populations [13–15], but have not yet been studied in European populations.

Altogether, adherence to several diet scores has been repeatedly studied in relation to CVD incidence and mortality [5, 16]. Findings have also highlighted that a 20-percentile increase in diet scores, including DASH and AHEI-2010, was associated with a significantly 3–9% lower risk of CVD in subsequent time-periods [17]. However, the long-term impact of diet scores on novel cardiovascular risk markers that are independent predictors of CVD events, e.g., augmentation index (AIx) and aortic pulse wave velocity (aPWV) [18], is still unclear. A previous study has shown that healthy lifestyle factors, including high fruit and vegetable consumption, were associated with reduced aPWV [19]; however, the association with diet as a whole, as measured by diet scores, is still unknown. The aim of the present study was to investigate whether adherence to a healthy diet (as assessed by the HDI, the DASH score, and the AHEI-2010) is associated CVD incidence, and to study the cross-sectional and longitudinal relationship with major CVD risk markers (both traditional and novel) in a community-based cohort of middle-aged men enrolled in the Caerphilly Prospective Study (CaPS) beginning in the late 1970s.

## Methods

### Study design and study population

The CaPS was established to investigate the importance of lipids, haemostatic factors, and hormones in the development of coronary heart disease (CHD) [20]. Initial 2512 men, aged 45–59 years from the town of Caerphilly and adjoining villages, South Wales (United Kingdom) were recruited (response rate 89%) and examined between 1979 until 1983 (Phase 1). Subsequent data-collection phases were undertaken at 5-year intervals: 1984–1988 (Phase 2), 1989–1993 (Phase 3), 1993–1997 (Phase 4), and 2002–2005 (Phase 5). Additional 447 men, aged 50–64 years, were included at Phase 2 as a result of 561 men being lost to follow-up, leaving a new total of 2398 men, and therefore, the present study considered Phase 2 as baseline. As outlined below, the exposure period is from Phase 2 (1984–1988) until Phase 3 (1989–1993). Up to Phase 4 (i.e. before 1993), 244 men who died, 159 men who had a history of myocardial infarction or stroke, and 116 men who had diabetes were excluded from the analyses. In addition, 12 men with missing dietary data to calculate their diet scores were excluded, leaving 1867 men for analyses. Written informed consent was obtained from all individual subjects included in the study, and the study had the approval of the local research ethics committee and adhered to the Declaration of Helsinki.

### Exposure assessment

After staff instruction, self-administered, semi-quantitative food frequency questionnaires (FFQ) were completed at home during Phase 2 and/or Phase 3. This questionnaire provided information on the frequency of 12 main food groups typical of the British diet (bread, breakfast cereals, meat, fish, vegetables, biscuits and puddings, fresh fruit, eggs, milk, fats, drinks, and alcohol), and this allowed an estimation of the average daily consumption of 50 food items. Validation and calculation of nutrient intakes of the FFQ have previously been described in detail [21, 22]. For the present study, mean dietary intakes over the exposure period (i.e., Phase 2 and Phase 3) were calculated to better account for the variation in dietary intake over time, and adherence to a healthy diet was assessed using existing diet scoring models (Table 1).

**Table 1:** Components and scoring of the diet scores

Diet scores and components	Scoring	
	Minimum score	Maximum score
Healthy diet indicator (HDI)^a^	0 points	1 point
Saturated fatty acids	≥10% total energy	<10% total energy
Polyunsaturated fatty acids	<6 or >10% total energy	6–10% total energy
Cholesterol	≥300 mg/day	<300 mg/day
Protein	<10 or >15% total energy	10–15% total energy
Fibre	<25 g/day	≥25 g/day
Free sugars	≥10 % total energy	<10% total energy
Fruits and vegetables	<400 g/day	≥400 g/day
Dietary approaches to stop hypertension (DASH)^b^	1 point	5 points
Fruits	Quintile 1	Quintile 5
Vegetables	Quintile 1	Quintile 5
Legumes	Quintile 1	Quintile 5
Whole grains	Quintile 1	Quintile 5
Milk	Quintile 1	Quintile 5
Sodium	Quintile 5	Quintile 1
Red and processed meat	Quintile 5	Quintile 1
Sugar-sweetened beverages	Quintile 5	Quintile 1
Alternative Healthy Eating Index (AHEI-2010)^c^	0 points	10 points
Vegetables	No intake	≥5 servings/day
Fruits	No intake	≥4 servings/day
Whole grains	No intake	≥6 servings/day
Legumes	No intake	≥1 serving/day
Oily fish	No intake	≥2 servings/week
Polyunsaturated fatty acids	≤2% total energy	≥10% total energy
Red and processed meat	≥1.5 servings/day	No intake
Trans fatty acids	≥4% total energy	≤0.5% total energy
Sodium	Highest decile	Lowest decile
Sugar-sweetened beverages	≥1 serving/day	No intake
Alcohol	≥3.5 drinks/day	0.5–2.0 drinks/day

^a^Scores for the components of the HDI on a dichotomous scale (0 or 1 point)

^b^Components’ scoring (ranging from 1 to 5 points) depends on the quintiles (1, 2, 3, 4, or 5 points), with higher quintiles representing higher intakes

^c^Scores for the components of the AHEI-2010 index on a continuous scale ranging from 0 to 10 points, with proportional scores for intermediate intakes (0, 1, 2, 3, 4, 5, 6, 7, 8, 9, or 10 points). Serving sizes are defined as follows: vegetables and fruits, 125 g; whole grains, 15 g; legumes, 100 g; oily fish, and red and processed meat, 115 g; sugar-sweetened beverages, 225 g; and alcohol, alcoholic drink containing 5 g pure ethanol

The HDI is based on the dietary guidelines for the prevention of chronic diseases defined by the WHO, published in 2003 [8]. The scoring criteria consist of six nutrients and one food group component including saturated fatty acids, polyunsaturated fatty acids, cholesterol, protein, fibre, free sugars, and fruits and vegetables [10]. A dichotomous variable was created, indicating that an intake within the recommended range was assigned a score of one for that component, zero otherwise. The sum resulted in a range from 0 (minimal adherence) to 7 (maximal adherence).

The DASH score, measuring adherence to the DASH diet, is based on eight criteria: high intake of fruits, vegetables, nuts and legumes, whole grains and low-fat dairy products, and low intake of sodium, red and processed meats, and sweetened beverages [23]. For each component, subjects were classified into quintiles according to their intake. The quintile ranking was considered as the component score, ranging from 1 to 5 points (representing 1 for the lowest intake and 5 for the highest intake; however, the scoring was reversed for the components where low intake was desired). As the consumption of fat-reduced milk and other dairy products such as yoghurt was not assessed in the FFQ, the DASH component of ‘low-fat dairy products’ was modified to reflect milk consumption only, including milk intake in tea or coffee and with cereals, and milky drinks [24, 25]. The component of ‘nuts and legumes’ represented only the intake of legumes as nut intake was not assessed in this study. The sum of the component scores resulted in an overall DASH score range from 8 (minimal adherence) to 40 (maximal adherence).

The AHEI-2010 consists of 11 components, of which six focus on adequacy of the diet (dietary components to increase, including vegetables, fruits, whole grains, nuts and legumes, long-chain omega-3 fatty acids, and polyunsaturated fatty acids) and the remaining five focus on moderation (dietary components to decrease, including sugar-sweetened beverages and fruit juices, red and processed meat, trans fatty acids, sodium, and alcohol) [13]. Similarly, the component of ‘nuts and legumes’ represented legumes only, and the component of ‘long-chain omega-3 fatty acids’ was changed into the intake of oily fish. For all components, a higher score reflects better adherence that is an intake at the level of the standard recommendation or higher received the maximum number of points, i.e., 10, for the adequacy components; otherwise, a proportionately lower score was assigned, with a score of zero as minimum. This scoring approach was reversed for the moderation components. The sum resulted in an overall score range from 0 (minimal adherence) to 110 (maximal adherence). More details about the components of the diet scores are shown in Online Resource 1.

### Covariates

The general questionnaires filled out by the subjects provided information on demographics, general health, and medical history regarding the presence of chronic diseases and risk factors or risk symptoms for CVD. Smoking status was categorised as never, former or current smoker. Social class was categorised as non-manual (including professional, managerial, and non-manual occupations) or manual (including manual, semi-skilled, and unskilled occupations). Physical activity, which was only measured at Phase 2, was categorised based on energy expenditure during leisure time activities as inactive, moderately inactive, moderately active, and active. Alcohol consumption was categorised as non-drinking, moderate drinking (≤20 g ethanol/day), or high drinking (>20 g ethanol/day). Phase 2 measurements were considered as the baseline values; however, when the values of Phase 2 covariates were missing, then those values were replaced by those measured at Phase 3. Missing values for smoking habits (0.3%) and social class (0.3%) at Phase 2 were replaced by those measured at Phase 3 (or Phase 1) as a proxy for the Phase 2 measurement.

### Cardiovascular risk markers

Body mass index (BMI) was calculated as weight (kg) divided by height (m) squared, which was measured at physical examinations during Phase 2 and Phase 3. During these examinations, resting systolic and diastolic BP were measured in duplicate at room temperature on the left upper arm, while the subject was seated using a Hawkslet random 0 sphygmomanometer at Phase 2 and Phase 3, and a validated Omron-705CP at phase 5 [26]. At Phase 5, AIx and aPWV were measured [27]. The AIx was derived from applanation of the radial artery, while the aPWV was calculated using applanation of the carotid and femoral arteries, using a validated SphygmoCor device [28]. Measurements were made in duplicate by a single operator after subjects had refrained from eating, drinking, and smoking for ≤3 h period [29]. At Phase 2 and Phase 3, fasting blood samples were taken for analysis of serum total cholesterol, triacylglycerol, and glucose. HDL and LDL cholesterol (calculated using the Friedewald formula [30]), and high-sensitivity C-reactive protein (CRP) were measured at Phase 2 only. Phase 5 blood assays were more limited; for that reason, only a Phase 5 equivalent measure was available for triacylglycerol and CRP. Details of the methods have been reported elsewhere [31]. Mean Phase 2 and Phase 3 variables were generated for BMI, systolic and diastolic BP, total cholesterol, triacylglycerol, and glucose to adjust for relevant confounders in the relationship with cardiovascular events, and subsequently, hypertension, dyslipidaemia, and impaired fasting glycaemia were defined as described in the European Guidelines on CVD prevention [32]. At Phase 2, an estimation of 10-year CVD risk was predicted by the Framingham Risk Score (FRS) for global CVD risk, including age, systolic BP not treated, total and HDL cholesterol, and smoking status [33].

### Verification of outcome

Incidence of cardiovascular events was continued until September 2014 and confirmed through primary care records, hospital records, and the National Health Service Central Registry that also kept death certificates coded by the 9th revision of the International Classification of Diseases (ICD). Clinical endpoints for the present analyses were incidence of CHD, including ischaemic heart diseases, cardiac arrest and sudden death (ICD-9 codes: 410–414, 427.5, 798.1, 798.2, 798.9), stroke (ICD-9 codes: 430–434, 436), and CVD, including CHD, stroke, and congestive heart failure (ICD-9 codes: 428), and both fatal and non-fatal events.

### Statistical analysis

#### Diet scores versus cardiovascular events

Cox proportional hazards analyses were performed to compute hazard ratios (HR) and the 95% confidence intervals (CI) for the relationship between adherence to a healthy diet and incidence of CVD, CHD, and stroke. Subjects were classified into tertiles depending on their diet score, using the lowest tertile (lowest diet score) as reference. In addition, analyses were repeated in which the diet score was modelled as a continuous variable, in particular a unit increment of one standard deviation of the mean diet score.

The duration of follow-up was defined as the time starting from after Phase 3 measurements were taken and onwards until the onset of CVD event, or censoring (mortality from another cause of death, loss to follow-up or final follow-up assessment in September 2014), whichever came first. The proportional hazard assumption was met, confirmed by graphical evaluations of log-minus-log plots. Estimates were adjusted for Phase 2 age (continuous), Phase 2 smoking status and intensity (categories), Phase 2 social class (categories), Phase 2 physical activity level (categories), mean Phase 2 and Phase 3 energy intake (continuous), and mean Phase 2 and Phase 3 usual alcohol consumption (categories). Alcohol consumption was left out the model, when it was included as a component of the diet score.

Incremental models, including mean Phase 2 and Phase 3 BMI (≥25 or <25 kg/m^2^), hypertension (yes or no), systolic and diastolic BP (continuous), dyslipidaemia (yes or no), impaired fasting glycaemia (yes or no), diabetes (yes or no), and CRP (continuous), separately in the multivariable model, were performed to study the possible intermediating role of these covariates in the relationship between diet and CVD events. Sensitivity analyses were conducted to determine how associations between diet scores and CVD outcomes changed when subjects with indication for CVD (history of angina, chest pain, and clinical or ECG evidence of infarction or stroke) were excluded from the analyses. However, given that the majority of the middle-aged men (67.9%) were preclinical patients, analyses were repeated including these subjects only.

In addition, the association between changes in diet scores and incidence of CVD, CHD, and stroke was investigated, in which changes in diet scores were divided into tertiles using the second tertile (relatively no change) as reference, and were used continuous as a unit increment of one standard deviation of the mean change in diet score. Included were 1713 subjects of whom complete dietary data at Phase 2 and Phase 3 were available. Estimates were adjusted for similar lifestyle factors as stated above and for the initial diet score.

#### Diet scores versus risk markers

Linear regression analyses were performed to further explore the association between adherence to a healthy diet and CVD risk markers, both cross-sectional (mean Phase 2 and 3 dietary intakes with mean Phase 2 and Phase 3 risk markers) and longitudinal (mean Phase 2 and 3 dietary intakes with Phase 5 risk markers). Estimates were adjusted for similar lifestyle factors as stated above, with an additional adjustment for BMI. Linear regression assumptions were checked by graphs of outcome variable versus explanatory variables and normal probability plots, and any deviations against normality were fixed by using log transformations.

A two-sided *P* value below 0.05 was considered as statistically significant. All analyses were carried out within the statistical software programme STATA, version 14 (STATA Corp, College Station, TX).

## Results

### Descriptive statistics

During a mean follow-up of 16.6 ± 7.2 years, 725 CVD events (509 non-fatal and 216 fatal) were identified in the total cohort of 1867 men, resulting in a CVD incidence rate of 23 per 1000 person-years. Of these CVD events, 407 CHD events (246 non-fatal and 161 fatal) and 209 stroke events (182 non-fatal and 27 fatal) were observed. Subjects with higher diet scores were less likely to be current smokers, more likely to have non-manual work, a lower alcohol consumption, and a lower FRS at baseline compared with the lowest scores (Table 2).

**Table 2:** Descriptive characteristics per diet score tertile of the 1867 middle-aged men in the Caerphilly Prospective Study at Phase 2

	Healthy diet indicator (score range 0–6)			Dietary approaches to stop hypertension score (score range 11–35)			Alternative healthy eating index-2010 (score range 10–80)		
	T1 (*n* = 854)	T2 (*n* = 619)	T3 (*n* = 394)	T1 (*n* = 550)	T2 (*n* = 604)	T3 (*n* = 713)	T1 (*n* = 610)	T2 (*n* = 624)	T3 (*n* = 633)
Diet score	0.8 ± 0.4	2 ± 0	3.3 ± 0.6	18.9 ± 2.0	23.5 ± 1.1	28.6 ± 2.3	27.1 ± 4.8	38.4 ± 2.9	51.3 ± 6.1
Follow-up (years)	16.1 ± 7.4	16.9 ± 6.8	17.3 ± 7.0	15.1 ± 7.5	16.6 ± 7.1	17.8 ± 6.8	15.5 ± 7.2	16.6 ± 7.2	17.7 ± 6.9
Age (years)	56.7 ± 4.6	56.5 ± 4.5	56.8 ± 4.2	56.5 ± 4.6	56.8 ± 4.4	56.6 ± 4.5	56.5 ± 4.7	56.7 ± 4.4	56.8 ± 4.4
Current smoking [*N*, (%)]	426 (49.9)	268 (43.3)	111 (28.2)	340 (61.8)	259 (42.9)	206 (28.9)	357 (58.5)	265 (42.5)	183 (28.9)
Non-manual worker [*N*, (%)]	260 (30.4)	210 (33.9)	159 (40.4)	122 (22.2)	178 (29.5)	329 (46.1)	132 (21.6)	224 (35.9)	273 (43.1)
Physically active [*N*, (%)]	383 (44.8)	281 (45.4)	155 (39.3)	233 (42.4)	270 (44.7)	316 (44.3)	258 (42.3)	279 (44.7)	282 (44.5)
Body mass index (kg/m^2^)	26.3 ± 3.7	26.5 ± 3.6	26.6 ± 3.3	25.5 ± 3.5	26.6 ± 3.6	27.1 ± 3.4	25.7 ± 3.6	26.6 ± 3.6	27.0 ± 3.4
Systolic blood pressure (mmHg)	145.0 ± 20.2	145.0 ± 19.4	145.5 ± 19.7	145.3 ± 19.7	145.9 ± 20.1	144.4 ± 19.8	145.4 ± 20.1	145.5 ± 19.7	144.4 ± 19.8
Diastolic blood pressure (mmHg)	82.8 ± 10.7	83.5 ± 9.9	83.1 ± 10.1	82.6 ± 10.7	83.5 ± 10.2	83.1 ± 10.1	83.0 ± 10.6	83.5 ± 10.4	82.7 ± 9.9
Total cholesterol (mmol/L)	5.9 ± 1.0	6.0 ± 1.0	5.9 ± 1.0	6.0 ± 1.0	5.9 ± 1.0	6.0 ± 1.0	6.0 ± 1.0	5.9 ± 1.0	6.0 ± 1.0
LDL cholesterol (mmol/L)	4.2 (0.9)	4.2 ± 1.0	4.2 ± 0.9	4.2 ± 0.9	4.2 ± 0.9	4.2 ± 1.0	4.2 ± 0.9	4.2 ± 0.9	4.3 ± 0.9
HDL cholesterol (mmol/L)	1.0 ± 0.2	1.0 ± 0.3	1.0 ± 0.2	1.0 ± 0.3	1.0 ± 0.2	1.0 ± 0.2	1.1 ± 0.3	1.0 ± 0.2	1.0 ± 0.2
Triacylglycerol (mmol/L)	1.7 (1.2–2.3)	1.7 (1.2–2.3)	1.6 (1.2–2.2)	1.7 (1.2–2.3)	1.7 (1.2–2.3)	1.6 (1.2–2.2)	1.7 (1.3–2.4)	1.6 (1.2–2.2)	1.6 (1.2–2.2)
Glucose (mmol/L)	5.2 ± 0.8	5.2 ± 0.8	5.2 ± 0.6	5.2 ± 0.9	5.2 ± 0.8	5.2 ± 0.6	5.2 ± 0.8	5.2 ± 0.9	5.2 ± 0.6
C-reactive protein (mg/L)	1.7 (0.9–3.3)	1.6 (0.8–3.6)	1.2 (0.7–2.6)	1.9 (1.0-3.7)	1.6 (0.8–3.4)	1.3 (0.6–2.7)	1.8 (0.9–3.6)	1.5 (0.8–3.1)	1.4 (0.7–2.9)
Framingham Risk Score (%)	27.9 ± 13.8	27.2 ± 13.8	25.3 ± 12.3	29.5 ± 14.4	27.1 ± 13.1	25.3 ± 12.9	28.8 ± 14.4	27.1 ± 13.2	25.6 ± 12.8
Dietary intake									
Total energy intake (kcal/day)	2150.7 ± 488.8	2041.0 ± 467.8	1940.4 ± 454.7	2336.0 ± 463.3	2070.8 ± 455.9	1864.0 ± 411.7	2352.9 ± 472.3	2079.7 ± 403.0	1787.7 ± 391.0
Fat (g/day)	82.8 ± 23.6	77.7 ± 24.2	71.2 ± 23.9	87.7 ± 25.0	79.0 ± 23.1	71.4 ± 22.2	86.4 ± 25.4	80.8 ± 22.6	69.1 ± 21.5
Carbohydrates (g/day)	251.6 ± 70.6	243.2 ± 64.2	238.3 ± 62.6	281.2 ± 67.5	244.8 ± 67.0	219.9 ± 53.3	278.1 ± 69.8	248.0 ± 61.0	213.2 ± 53.4
Protein (g/day)	74.8 ± 13.7	70.2 ± 15.4	69.3 ± 15.0	75.9 ± 14.2	71.5 ± 15.3	69.7 ± 14.2	76.4 ± 14.3	72.1 ± 14.9	68.0 ± 13.9
Fibre (g/day)	18.4 ± 4.6	20.4 ± 6.3	25.8 ± 7.3	17.1 ± 4.2	19.7 ± 5.9	24.2 ± 6.7	17.4 ± 4.6	20.2 ± 5.9	24.2 ± 6.8
Sodium (mg/day)	2372.2 ± 577.5	2284.8 ± 644.1	2297.8 ± 666.6	2575.0 ± 596.7	2329.6 ± 611.9	2134.8 ± 577.3	2525.6 ± 607.7	2323.6 ± 605.9	2140.5 ± 588.5
Cholesterol (mg/day)	378.9 ± 96.3	321.1 ± 96.6	289.0 ± 88.4	378.2 ± 101.6	344.0 ± 102.1	309.2 ± 90.7	381.6 ± 107.0	343.0 ± 95.1	299.3 ± 85.1
Vegetable intake (g/day)	145.1 (107.1–187.5)	147.3 (102.7–205.4)	189.7 (138.4–241.1)	120.5 (89.3–151.8)	151.8 (107.1–196.4)	196.4 (142.9–241.1)	138.4 (93.8–178.6)	147.3 (107.1–196.4)	178.6 (133.9–232.1)
Fruit intake (g/day)	103.6 (49.1–171.4)	133.9 (58.0–212.5)	96.4 (178.6–285.7)	62.5 (22.3–112.5)	116.1 (62.5–178.6)	196.4 (129.5–282.1)	71.4 (26.8–133.9)	125.0 (67.0–196.4)	186.6 (116.1–271.4)
Meat intake (g/day)	135.5 (106.8–176.6)	115.0 (82.1–156.1)	102.7 (73.9–139.6)	152.0 (119.1–188.9)	127.3 (94.5–164.3)	102.7 (73.9–131.4)	152.0 (115.0–188.9)	90.4 (119.1–156.1)	102.7 (69.8–135.5)
Ethanol intake (g/day)	11.4 (2.8–25.2)	10.1 (2.0–20.2)	10.1 (1.9–18.9)	13.9 (3.6–26.1)	11.4 (2.0–22.7)	7.7 (1.8–17.7)	20.1 (8.9–35.3)	10.1 (1.9–20.2)	6.4 (1.8–12.7)

Data are presented as *N* with %, mean with standard deviation or as median with interquartile range when the variable was not normally distributed

### Diet scores versus cardiovascular events

The HDI was not associated with CVD incidence after multivariable adjustment (Table 3) when comparing subjects with the highest tertile of HDI scores with the lowest (HR 0.89; 95% CI 0.73, 1.10) or when analysed per standard deviation increase in score (HR 0.98; 0.91, 1.06). Similarly, no relations with CHD and stroke incidence were found.

**Table 3:** Hazard ratios with 95% CIs for cardiovascular disease-coronary heart disease and stroke by diet scores in the Caerphilly Prospective Study

	Cardiovascular disease			Coronary heart disease			Stroke		
	Events	Crude HR (95% CI)	Adjusted HR^a^ (95% CI)	Events	Crude HR (95% CI)	Adjusted HR^a^ (95% CI)	Events	Crude HR (95% CI)	Adjusted HR^a^ (95% CI)
Healthy diet indicator									
T1 (*n* = 854)	344	1.00 (Referent)	1.00 (Referent)	194	1.00 (Referent)	1.00 (Referent)	105	1.00 (Referent)	1.00 (Referent)
T2 (*n* = 619)	243	0.94 (0.80–1.11)	1.01 (0.8–1.19)	139	0.95 (0.77–1.19)	1.00 (0.81–1.25)	67	0.85 (0.62–1.15)	0.94 (0.68–1.28)
T3 (*n* = 394)	138	0.78 (0.64–0.95)	0.89 (0.73–1.10)	74	0.75 (0.57–0.98)	0.86 (0.65–1.13)	37	0.68 (0.47–0.99)	0.78 (0.53–1.14)
Per SD (1.0)	725	0.94 (0.88–1.01)	0.98 (0.91–1.06)	407	0.93 (0.84–1.02)	0.97 (0.88–1.07)	209	0.92 (0.80–1.05)	0.96 (0.83–1.10)
Dietary approaches to stop hypertension score									
T1 (*n* = 550)	227	1.00 (Referent)	1.00 (Referent)	121	1.00 (Referent)	1.00 (Referent)	76	1.00 (Referent)	1.00 (Referent)
T2 (*n* = 604)	228	0.80 (0.67–0.96)	0.85 (0.70–1.03)	138	0.91 (0.72–1.17)	0.97 (0.75–1.25)	60	0.63 (0.45–0.88)	0.66 (0.46–0.94)
T3 (*n* = 713)	270	0.70 (0.58–0.83)	0.81 (0.66–0.99)	148	0.73 (0.57–0.92)	0.84 (0.64–1.10)	73	0.55 (0.40–0.76)	0.61 (0.42–0.88)
Per SD (4.4)	725	0.86 (0.80–0.92)	0.92 (0.84-1.00)	407	0.86 (0.78–0.95)	0.91 (0.81–1.02)	209	0.81 (0.70–0.92)	0.85 (0.73-1.00)
Alternative healthy eating index-2010^b^									
T1 (*n* = 610)	238	1.00 (Referent)	1.00 (Referent)	135	1.00 (Referent)	1.00 (Referent)	72	1.00 (Referent)	1.00 (Referent)
T2 (*n* = 624)	249	0.88 (0.74–1.06)	0.94 (0.78–1.14)	135	0.85 (0.67–1.08)	0.87 (0.68–1.12)	79	0.93 (0.67–1.27)	1.00 (0.71–1.41)
T3 (*n* = 633)	238	0.74 (0.62–0.88)	0.82 (0.66–1.01)	137	0.76 (0.60–0.96)	0.80 (0.61–1.06)	58	0.58 (0.41–0.82)	0.66 (0.44–0.99)
Per SD (11.0)	725	0.88 (0.82–0.96)	0.93 (0.85–1.02)	407	0.90 (0.82-1.00)	0.94 (0.83–1.06)	209	0.79 (0.69–0.91)	0.83 (0.70–0.98)

*HR* hazard ratio-*CI* confidence intervals

^a^Adjusted for age-smoking habits-social class-physical activity-total energy intake and usual alcohol consumption

^b^No adjustment for usual alcohol consumption in the multivariable model

There was a significant inverse association between the DASH score and CVD and stroke incidence after adjustment when comparing the highest tertile of DASH scores with the lowest (HR 0.81; 0.66, 0.99 and HR 0.61; 0.42, 0.88, respectively). When analysed per standard deviation increase in score, these associations were only borderline significant after multivariable adjustment (*P* = 0.053 and *P* = 0.054 respectively). No significant associations with CHD were observed.

A higher AHEI-2010 score was significantly associated with lower stroke incidence after adjustment when comparing the highest tertile with the lowest (HR 0.66; 0.44, 0.99) and when analysed per standard deviation increase in score (HR 0.83; 0.70, 0.98). The association with CVD was only borderline significant when comparing the highest tertile with the lowest (HR 0.82; 0.66, 1.01; *P* = 0.059), and no significant associations with CHD were observed.

When considering changes in diet scores, an increase in DASH score and AHEI-2010 was significantly associated with a decreased risk for stroke incidence when analysed per standard deviation increase in diet score change (HR 0.77; 0.66, 0.89 and HR 0.80; 0.68, 0.93, respectively). However, when analysed categorically, only a decrease in DASH score and AHEI-2010 was significantly associated with an increased risk of stroke (HR 1.39; 0.99, 1.98 and HR 1.42; 1.00, 2.03) (Online Resources 2).

### Diet scores versus risk markers: cross-sectional association

Results of the cross-sectional relationship between diet scores and CVD risk markers are presented in Online Resource 3. The HDI index was only significantly associated with CRP after adjustment for all known confounding variables; subjects with the highest HDI scores had a CRP level that was 18% (95% CI 4, 32) lower compared with subjects with the lowest HDI scores.

Higher DASH scores were significantly associated with higher BMI and lower CRP; when subjects with the highest DASH score were compared with subjects with the lowest DASH score, BMI was 0.99 kg/m^2^ (95% CI 0.56, 1.43) higher and CRP was 27% (95% CI 13, 42) lower. No clear associations with the remaining risk markers were observed.

Regarding the AHEI-2010, subjects with the highest scores had a significant 0.54 kg/m^2^ (95% CI 0.09, 0.99) higher BMI, a significant 1.47 mmHg (95% CI 0.18, 2.77) lower diastolic BP, a significant 0.04 mmol/L (95% CI 0.01, 0.07) lower HDL cholesterol, a significant 11% (95% CI 5, 16) lower triacylglycerol, and a significant 20% (95% CI 5, 34) lower CRP level compared with subjects with the lowest AHEI-2010 scores after confounding adjustment. No clear associations with the other risk markers were found.

### Diet scores versus risk markers: longitudinal association

The longitudinal associations between higher diet scores and CVD risk markers, representing a follow-up period of 11.8 years (SD 1.1), were based on 766 subjects of whom data on Phase 5 measurements were available (Table 4). No clear associations with HDI were found.

**Table 4:** Longitudinal relationship between diet scores (mean Phase 2 and Phase 3), and cardiovascular risk markers (Phase 5) in the Caerphilly Prospective Study

	Healthy diet indicator				Dietary approaches to stop hypertension score				Alternative healthy eating index-2010^a^			
	T1	T2	T3	Per SD	T1	T2	T3	Per SD	T1	T2	T3	Per SD
Systolic blood pressure (mmHg)												
Crude	Referent	−2.54 (−5.80, 0.72)	−1.37 (−4.91, 2.17)	−0.52 (−1.85, 0.81)	Referent	−2.90 (−6.74, 0.94)	−1.37 (−5.10, 2.27)	−0.14 (−1.56, 1.29)	Referent	−1.63 (−5.25, 1.99)	−1.87 (−5.39, 1.66)	−0.91 (−2.33, 0.52)
Adjusted	Referent	−2.00 (−5.30, 1.30)	−0.98 (−4.58, 2.63)	−0.40 (−1.76, 0.95)	Referent	−3.27 (−7.26, 0.72)	−1.76 (−5.84, 2.32)	−0.13 (−1.76, 1.49)	Referent	−1.69 (−5.45, 2.08)	−1.84 (−5.89, 2.21)	−1.07 (−2.75, 0.61)
Diastolic blood pressure (mmHg)												
Crude	Referent	−0.50 (−2.34, 1.34)	−0.40 (−2.40, 1.60)	−0.04 (−0.79, 0.71)	Referent	−0.94 (−3.10, 1.23)	−1.62 (−3.67, 0.44)	−0.78 (−1.58, 0.02)	Referent	−0.68 (−2.72, 1.36)	−1.52 (−3.51, 0.47)	−0.79 (−1.60, 0.01)
Adjusted	Referent	−0.66 (−2.51, 1.20)	−0.07 (−2.10, 1.95)	0.11 (−0.66, 0.87)	Referent	−1.03 (−3.27, 1.21)	−1.94 (−4.23, 0.35)	−0.94 (−1.85, −0.04)*	Referent	−0.86 (−2.96, 1.25)	−1.56 (−3.82, 0.71)	−0.87 (−1.81, 0.08)
Augmentation index (%)												
Crude	Referent	−1.45 (−2.99, 0.08)	−0.95 (−2.61, 0.72)	−0.43 (−1.06, 0.20)	Referent	−1.69 (−3.49, 0.12)	−1.00 (−2.71, 0.72)	−0.32 (−0.99, 0.35)	Referent	−0.08 (−1.78, 1.63)	0.51 (−1.16, 2.17)	0.05 (−0.63, 0.72)
Adjusted	Referent	−1.27 (−2.84, 0.29)	−0.72 (−2.42, 0.99)	−0.37 (−1.02, 0.27)	Referent	−1.61 (−3.50, 0.28)	−0.73 (−2.66, 1.20)	−0.20 (−0.97, 0.57)	Referent	0.22 (−1.56, 2.00)	1.36 (−0.55, 3.27)	0.35 (−0.44, 1.15)
Pulse wave velocity (m/s)												
Crude	Referent	−0.27 (−0.73, 0.20)	−0.14 (−0.64, 0.37)	−0.02 (−0.21, 0.17)	Referent	−0.06 (−0.61, 0.49)	−0.32 (−0.84, 0.20)	−0.14 (−0.35, 0.06)	Referent	−0.24 (−0.76, 0.27)	−0.58 (−1.09, −0.07)	−0.24 (−0.44, −0.04)**
Adjusted	Referent	−0.15 (−0.59, 0.30)	−0.13 (−0.62, 0.35)	−0.03 (−0.21, 0.15)	Referent	−0.25 (−0.79, 0.28)	−0.51 (−1.06, 0.03)	−0.19 (−0.41, 0.02)	Referent	−0.37 (−0.88, 0.13)	−0.77 (−1.31, −0.23)*	−0.36 (−0.58, −0.14)**
Log triacylglycerol (mmol/L)												
Crude	Referent	0.05 (−0.03, 0.13)	0.01 (−0.08, 0.10)	0.01 (−0.02, 0.04)	Referent	0.02 (−0.08, 0.11)	−0.03 (−0.12, 0.06)	−0.04 (−0.07, 0.00)	Referent	−0.01 (−0.10, 0.08)	−0.04 (−0.13, 0.04)	−0.04 (−0.08, −0.01)*
Adjusted	Referent	0.05 (−0.03, 0.13)	0.03 (−0.06, 0.12)	0.02 (−0.02, 0.05)	Referent	0.03 (−0.07, 0.13)	0.02 (−0.08, 0.12)	−0.02 (−0.06, 0.02)	Referent	−0.001 (−0.09, 0.09)	−0.001 (−0.10, 0.10)	−0.03 (−0.07, 0.01)
Log C—reactive protein (mg/L)												
Crude	Referent	0.00 (−0.21, 0.22)	−0.08 (−0.32, 0.15)	−0.03 (−0.11, 0.06)	Referent	−0.09 (−0.35, 0.16)	−0.21 (−0.45, 0.03)	−0.06 (−0.16, 0.03)	Referent	0.02 (−0.22, 0.25)	−0.17 (−0.40, 0.07)	−0.12 (−0.21, −0.02)**
Adjusted	Referent	−0.00 (−0.22, 0.21)	−0.02 (−0.26, 0.21)	−0.01 (−0.10, 0.07)	Referent	−0.04 (−0.30, 0.23)	−0.12 (−0.39, 0.14)	−0.03 (−0.14, 0.07)	Referent	0.02 (−0.23, 0.26)	−0.14 (−0.41, 0.11)	−0.11 (−0.22, 0.00)**

Crude and adjusted change in cardiovascular risk markers expressed using the regression coefficients (95% CI). Adjusted for age, smoking status, social class, physical activity, total energy intake, usual alcohol consumption, and BMI

*P < 0.01 and *P* trend < 0.01; **P < 0.05

^a^No adjustment for usual alcohol consumption in the multivariable model

After multivariable adjustment, the DASH score was significantly associated with diastolic BP when analysed continuously; diastolic BP was 0.9 mmHg (95% CI 0.04, 1.85) lower on average with each standard deviation increase in score. However, when comparing subjects in the highest tertile scores with the lowest, a borderline significant association with aPWV was found (*P* = 0.064).

Higher AHEI-2010 scores were significantly associated with lower arterial stiffness (measured by aPWV); when comparing subjects in the highest tertile scores with the lowest, aPWV was 0.77 m/s (95% CI 0.23, 1.31) lower, and when analysed per standard deviation increase aPWV was 0.36 m/s (95% CI 0.14, 0.58) lower on average after adjustment. In addition, higher AHEI-2010 scores were significantly associated with lower CRP levels when analysed continuously, in particular after back transformation to the original scale, CRP levels were 11% (95% CI 1, 20) lower on average with each standard deviation increase in score. No clear associations were found for the remaining risk markers, except for diastolic BP that was borderline significant when analysed continuously (*P* = 0.071).

### Sensitivity analyses

Incremental models, including BMI, hypertension, dyslipidaemia, impaired fasting glycaemia, or diabetes in the multivariate Cox model, to explore the possible mechanisms through various risk markers in the relationship between overall diet and CVD outcomes did not alter the results (data not shown). However, including CRP as a mediator in the multivariable model did change the results, especially the association with CHD incidence was attenuated after inclusion of CRP for the DASH score (HR 0.84; 0.64, 1.10 and after inclusion of CRP HR 0.96; 0.83, 1.28), while the association with stroke incidence became stronger after inclusion of CRP for the AHEI-2010 (HR 0.66; 0.44, 0.99 and after adjusting HR 0.56; 0.34, 0.93).

In this cohort, only 586 middle-aged men (31.4%) had no indication for preclinical cardiovascular disease at Phase 2 and Phase 3, of which 175 incident CVD cases (including 82 CHD events and 65 stroke events) were documented at a CVD incidence rate of 17 per 1000 person-years. Restricting the analyses to these subjects mostly yielded stronger associations between diet score and incidence outcomes under study (Online Resource 4). In this subpopulation, the DASH score was significantly stronger associated with a lower risk of CVD when comparing the highest tertile with the lowest (HR 0.64; 0.42, 0.96) and when analysed per standard deviation increase (HR 0.82; 0.69, 0.97). In addition, the DASH score was significantly stronger associated with a lower risk of stroke when analysed continuously (HR 0.76; 0.59, 1.00). For the AHEI-2010, higher scores were significantly associated with a lower risk of CVD, both when analysed in tertiles (HR 0.63; 0.41, 0.98), and when per standard deviation increase (HR 0.82; 0.68, 0.99) after multivariable adjustment. The association with stroke became only borderline significant when analysed per standard deviation increase in score (HR 0.74; 0.55, 1.00). In the sub-cohort of 1,268 preclinical patients (e.g., patients with a history of angina, chest pain, and clinical or ECG evidence of infarction or stroke), of which 545 incident CVD cases (including 321 CHD events and 143 stroke events) were documented at a CVD incidence rate of 26 per 1,000 person-years, no significant associations between diet scores and CVD or CHD incidence were observed, whereas the associations with stroke became significantly stronger for the HDI (HR 0.58; 0.35, 0.97) and the DASH score (HR 0.58; 0.37, 0.90) when comparing the highest tertile with the lowest (Online Resource 5).

## Discussion

In this longitudinal analysis (mean 16.6 ± 7.2 years) of 1,867 middle-aged men from the CaPS, we illustrated for the first time that diet scores are useful for CVD risk prediction in this particular population of middle-aged men, living in South Wales. We observed that higher DASH scores were associated with a lower CVD and stroke incidence, showing stronger associations when the study population was restricted to subjects without evidence for preclinical CVD at baseline. Higher scores on the AHEI-2010 were significantly associated with lower stroke incidence, while a significant association with CVD observed in subjects without preclinical CVD at baseline. No associations were found for CHD incidence. The main finding in the cross-sectional analyses indicated that higher diet scores were associated with lower CRP levels. These results were consistent for all three diet score indices under study, with the strongest association for the DASH score. However, the AHEI-2010 was inversely associated with diastolic BP, HDL cholesterol, and triacylglycerol after adjustment for possible confounding lifestyle factors and BMI. Importantly, after approximately 12 years of follow-up, the DASH score was also associated with lower diastolic BP, and the AHEI-2010 was associated with lower aPWV and lower CRP concentrations.

Similar to the present findings, literature on HDI in association with CVD incidence in Dutch populations [9] and older British men [34] also found no significant relationship, whereas a significant inverse association with CVD mortality in European populations has previously been observed [10, 35]. However, one study could only reveal this significant association when applying a continuous scoring method instead of the dichotomised scoring as used in the original HDI. Therefore, it is likely that continuous scoring might improve risk predictions, since this provides greater variation between subjects and represents the fact that intake levels rather than one specified cut-off value influence health outcomes of dietary components.

A recently published meta-analyses based on observational prospective studies showed that a higher adherence to the DASH diet was associated with a significant 20% CVD risk reduction and a lower risk for CHD or stroke incidence, although the results found for CHD and stroke should be interpreted with caution because of sensitivity to a single study [5, 36]. In contrast, the present study only observed a significant association with CHD incidence before adjustment for possible confounding lifestyle factors. This attenuation of the association was in line with findings from the Women Health Professional study, comparing associations of the DASH score with CVD risk [37]. In the present study, the modification of the DASH score with regard to the milk component as a proxy for low-fat dairy products might not have been entirely appropriate. It should be noted that the most common form of milk consumed by individuals would have been whole milk [24, 25] as opposed to low-fat milk or dairy that forms a component of the DASH score [23]. In recognition of this limitation, sensitivity analysis on the DASH score that excluded the milk component was conducted. This showed a slightly stronger association with CVD and stroke incidence, but still no association for CHD (Online Resource 6).

Published literature on the AHEI-2010 has shown a strong inverse association with CVD, CHD, and stroke incidence [5], which was not found in the current study. The lack of a significant association with CVD or CHD might be, because very few subjects in the total cohort (8%) had AHEI-2010 scores in the upper half of the scoring range, with low scores observed for the components that were potentially associated with CHD risk reduction, i.e., vegetables, fruits, legumes, oily fish, polyunsaturated fatty acids, and sugar-sweetened beverages. This is illustrated by the fact that mean AHEI-2010 scores observed in our study were around 51.3 for the upper tertile, whereas significant associations with CVD incidence were observed previously from a median AHEI-2010 score of 63.1 onwards in a middle-aged male population [13]. Another source of inconsistency might be due to the differences in sample size [13–15], population groups [14], and CVD incidence or mortality as outcome [14, 15]. Results of the present study on diet score and BMI do not support the findings of the previous cross-sectional studies, which have suggested that low adherence to a healthy diet is associated with overweight and obesity [38–40]. It is difficult to explain these results, but the possible influence of energy intake and physical activity levels before subjects were recruited into the CaPS, which cannot be ruled out. Subjects with higher diet scores tended to have lower energy intake without any differences in physical activity at Phase 2 measurements; however, this does not necessarily reflect past dietary exposures at younger age. This could be due to reverse causality where individuals who are overweight or obese are more likely to improve their diet and/or reduce their energy intake as a result of advice or motivation to change weight. Moreover, irrespective of BMI, higher diet scores were associated with a lower risk of developing CVD, since associations did not change after inclusion of BMI in the multivariate model. This corroborates the findings of a previous study which reported lower mortality risk among middle-aged subjects who were overweight [41].

The DASH score and the AHEI-2010 were inversely associated with diastolic BP at baseline and up to 12 years, implying that long-term adherence to a healthy diet is an effective nutritional strategy to improve BP. In addition, both scores were significantly associated with a lower stroke risk, suggesting a potential mechanism between diet and stroke through BP control [42]. However, this mediated effect by BP could not be confirmed, since including hypertension, systolic, and diastolic BP, separately, in the multivariable model did not alter the association between diet scores and stroke.

Previously, we have demonstrated that milk and dairy intake were associated with favourable influences on prospective arterial stiffness measures [43], which supports the inverse association between the DASH score, that contains dairy products, and aPWV observed in the present study. In addition, recent findings from the SU.VI.MAX study found that a nutritionally poor dietary pattern, characterised by high meat and alcohol consumption and low micronutrient intake, was associated with a slightly higher aPWV after a 7.5 year follow-up [44]. Both studies support the possible role of a healthy diet in the prevention of stiffening of large arteries, which might reduce CVD risk as aPWV is an independent predictor of CVD events and all-cause mortality [18].

Surprisingly, the DASH score, that is based on a diet to prevent hypertension [23], was only borderline significantly associated with aPWV, whereas the AHEI-2010 was significantly associated. This difference might be due to the method of scoring, in which the DASH score shows weaker sensitivity, because the individual score depends on the quintile component intakes of the population under study [45]. In addition, a previous small-scale study, investigating the relationship between DASH and peripheral vascular function, suggested that the BP lowering effect of the DASH diet is not mediated by alteration of peripheral vascular resistance [46].

In agreement with recent literature that demonstrated a cross-sectional inverse association between the AHEI-2010 and CRP [47], the cross-sectional analysis of the present study could confirm this association between diet scores and CRP, while the longitudinal analysis also found that higher AHEI-2010 scores were associated with lower CRP concentrations after a 12 year follow-up. Moreover, the associations between diet scores and CHD incidence were attenuated after inclusion of CRP in the multivariable model. This suggests a plausible mechanism by which chronic inflammation (elevated CRP) may in part mediate the relationship between diet and CHD [48]. However, further studies are required before drawing conclusions on causation.

The present study underscores the importance of reflecting on an individual’s diet, which is facilitated by the use of a diet score measuring the degree of adherence to a healthy diet. For a diet score to be predictive of disease risk, it is essential that it is regularly updated to reflect the current evidence based on diet–disease relationships [13]. Besides the inclusion of food groups and/or nutrients that are strongly associated with disease risk, an optimal diet score should evaluate the dietary intake against the recommendations using a continuous scoring scale. This would allow identification of high-risk individuals based on their dietary intake, as shown in this study, where a high DASH score and AHEI-2010 were clearly associated with a lower CVD risk. However, the feasibility in public health practice should still be explored, as diet scores do not merely include food groups and/or nutrients that are relatively simple to measure, i.e., types of fatty acid intake and sodium intake in the AHEI-2010.

The simple assessment of adherence to a healthy diet is still not considered as a practical tool for the stratification of CVD risk in the general population. Current risk stratification models quantify the traditional and novel physiological risk makers, with no considerations of dietary intake [49]. However, a healthy diet is recognised as a significant mediator of CVD risk reduction [5]. Findings from the present study suggest that assessing adherence to a healthy diet might also be valuable for identifying individuals at higher risk of CVD, as subjects with higher diet scores had a lower FRS at baseline and a favourable CVD risk profile after 12 years of follow-up. In addition, our results showed that an increase in adherence to a healthy diet was associated with a lower risk for stroke, which was also confirmed in a previous study [17]. Thus, a risk prediction model based on adherence to a healthy diet might improve the applicability in public health practice, since no physiological measurements have to be taken to estimate CVD risk and this may help to reduce barriers for the initial detection of CVD risk [50]. Further research is needed to confirm this finding.

Major strengths of the present study are its prospective design with over 10 years of follow-up for CVD risk markers and over 16 years of follow-up for CVD clinical endpoints. In addition, three diet scores were selected to explore the associations with CVD incidence and risk markers, and this allowed examination of the consistency of the findings across the scores. The dietary intake was assessed during Phase 2 and Phase 3, which made it possible to estimate long-term dietary intake more accurately. A potential limitation is that the assessment of dietary intake was based on the self-report using a semi-quantitative FFQ, which might have led to an over- or underestimation of the actual dietary intake. As a result, the allocating of the diet scores might be imprecise, but it is unlikely that the ranking of the subjects (with reference to the diet scores) is biased. It is of note that the FFQ was carefully validated against a 7-day weighed diet diary and found to be comparable [21, 22]. However, the non-differential random misclassification tends to suppress the estimates for the association between diet scores and health outcomes towards the null that might partly explain the relatively weak associations observed in the present study. Furthermore, as with any observational study, residual confounding could, in part, affect the associations observed due to possible measurement error in the self-reported covariates, including smoking status, physical activity, and alcohol intake. Another possible limitation is that the majority of the middle-aged men were preclinical patients, resulting in attenuation in the strength of association. Analyses in this subsample showed weaker associations compared to a subsample of healthy subjects, supporting the view that these subjects who had indication for preclinical CVD at baseline were more likely to improve their diet as a result of their condition, and that this reverse causation might have had a significant weakening impact on the association observed. Only a few subjects in this cohort consumed a diet that was close to the dietary recommendations for a healthy diet that might have limited the ability to observe significant associations with CVD incidence and risk markers. An issue that was not addressed in this study was that medication use and long-term changes to lifestyle, including dietary intake, physical activity, and smoking habits, also could have played a role in cardiovascular health. In addition, it was not possible to account for the development of cancer; therefore, it is unknown whether cancer treatment might have led to increased risk of CVD in long-term survivors [51]. It is acknowledged that there may be gender-specific differences in cardiovascular risk; further research is needed to verify whether these findings can be generalised to women [52]. These results might also not be applicable to non-Caerphilly populations.

In conclusion, in this cohort of middle-aged men, higher adherence to a healthy diet, as estimated by the DASH score and the AHEI-2010, was associated with a 20–40% lower risk of developing incident CVD and stroke. In addition, higher healthy diet scores were also associated with a favourable cardiovascular health status after 12 years of follow-up. Further studies are required to explore the relative value of diet scores in public health practice for the stratification of CVD risk in the general population.

## Electronic supplementary material

Below is the link to the electronic supplementary material.

Supplementary material 1 (DOCX 59 KB)

## Abbreviations

AHEI-2010: Alternative healthy eating index-2010
AIx: Augmentation index
aPWV: Aortic pulse wave velocity
BMI: Body mass index
BP: Blood pressure
CaPS: Caerphilly prospective study
CHD: Coronary heart diseases
CI: Confidence interval
CRP: High-sensitivity C-reactive protein
CVD: Cardiovascular diseases
DASH: Dietary approaches to stop hypertension
FFQ: Food frequency questionnaire
FRS: Framingham risk score
HDI: Healthy diet indicator
HR: Hazard ratio
ICD: International classification of diseases
SD: Standard deviation
WHO: World Health Organisation
