# Supplementary material for: Adherence to a healthy diet in relation to cardiovascular incidence and risk markers: evidence from the Caerphilly Prospective Study
Source: Eur J Nutr. 2017 Mar 14;57(3):1245–58. doi: 10.1007/s00394-017-1408-0 (PMC5861161; doi:10.1007/s00394-017-1408-0)
Supplement: Supplementary file 1 — Supplementary material 1 (DOCX 59 KB) [file 394_2017_1408_MOESM1_ESM.docx]

**Online Resource 1** Components of the diet scores with the foods included.

| Diet scores and components | Foods included |
| --- | --- |
| Healthy Diet Indicator (HDI) | |
| Saturated fatty acids  Polyunsaturated fatty acids  Cholesterol  Protein  Fibre  Fruits and vegetables  Free sugars | Sum of saturated fat content of all foods in FFQ  Sum of polyunsaturated fat content of all foods in FFQ  Sum of cholesterol content of all foods in FFQ  Sum of protein content of all foods in FFQ  Sum of fibre content of all foods in FFQ  All fruits and vegetables, excluding fruit juices, potatoes and legumes  Sum of all mono- and disaccharides added to foods by the manufacturer, cook or consumer, plus sugars naturally present in fruits, honey, syrups and fruit juices |
| Dietary Approaches to Stop Hypertension (DASH) score | |
| Fruits  Vegetables  Legumes  Whole grains  Milk products  Sodium  Red and processed meat  Sugar-sweetened beverages | All fruit and natural fruit juices  All vegetables, excluding potatoes and legumes  Baked beans, lentils or butter beans  Brown and whole meal breads, and whole grain breakfast cereals (such as muesli, shredded wheat, sultana Bran or Weetabix, bran flakes or puffed wheat and all bran or wheat bran)  Milk in tea or coffee, in milky drinks, or with cereals  Sum of sodium content of all foods in FFQ  Beef, lamb, pork or bacon or ham, tinned meat, pork and beef sausages, meat pies or pasties and organ meats (liver or kidney or heart)  Squashes, fizzy drinks, and tea or coffee with sugar |
| Alternative Healthy Eating Index (AHEI-2010) | |
| Vegetables  Fruits  Whole grains  Legumes  Oily fish  Polyunsaturated fatty acids  Red and processed meat  Trans-fatty acids  Sodium  Sugar-sweetened beverages Alcohol | All vegetables, excluding potatoes and legumes  All kinds of whole fruits, excluding 100% fruit juices  Brown and whole meal breads, and whole grain breakfast cereals (such as muesli, shredded wheat, sultana Bran or Weetabix, bran flakes or puffed wheat and all bran or wheat bran)  Baked beans, lentils or butter beans  Kippers, herring, pilchards, tuna, sardines, salmon or mackerel, including tinned oily fish products  Sum of polyunsaturated fat content of all foods in FFQ  Beef, lamb, pork or bacon or ham, tinned meat, pork and beef sausages, meat pies or pasties and organ meats (liver or kidney or heart)  Sum of trans-fatty acids content of all foods in FFQ  Sum of sodium content of all foods in FFQ  Fruit juices and squashes, plus sugar added in tea or coffee  Sum of the alcoholic drinks containing 5 g pure ethanol |

**Online Resources 2** Hazard ratios with 95 % CIs for cardiovascular disease-coronary heart disease and stroke by diet score changes in the Caerphilly Prospective Study.

|  |  | Cardiovascular disease | | | |  | Coronary heart disease | | | |  | Stroke | | | | |
| --- | --- | --- | --- | --- | --- | --- | --- | --- | --- | --- | --- | --- | --- | --- | --- | --- |
|  |  | Events | | Crude HR  (95 % CI) | Adjusted HR^a^ (95 % CI) |  | Events | Crude HR (95 % CI) | Adjusted HR^a^ (95 % CI) | |  | Events | Crude HR  (95 % CI) | | | Adjusted HR^a^ (95 % CI) |
| Healthy Diet Indicator | | | | | | | | | | | | | | | | |
| T1 (n=437) |  | | 169 | 1.00 (0.82-1.21) | 1.11 (0.90-1.38) |  | 97 | 0.99 (0.77-1.29) | | 1.11 (0.84-1.48) |  | 46 | 1.01 (0.69-1.47) | | 1.23 (0.81-1.86) | |
| T2 (n=638) |  | | 252 | 1.00 (Referent) | 1.00 (Referent) |  | 146 | 1.00 (Referent) | | 1.00 (Referent) |  | 67 | 1.00 (Referent) | | 1.00 (Referent) | |
| T3 (n=638) |  | | 248 | 0.92 (0.77-1.10) | 0.92 (0.77-1.10) |  | 135 | 0.87 (0.69-1.09) | | 0.87 (0.69-1.11) |  | 79 | 1.10 (0.79-1.52) | | 1.08 (0.77-1.51) | |
| Per SD (1.2) |  | | 669 | 0.97 (0.90-1.05) | 0.96 (0.87-1.04) |  | 378 | 0.94 (0.85-1.04) | | 0.93 (0.82-1.04) |  | 192 | 1.06 (0.92-1.22) | | 1.01 (0.86-1.20) | |
| Dietary Approaches to Stop Hypertension score | | | | | | | | | | | | | | | | |
| T1 (n=555) |  | 213 | | 1.01 (0.83-1.22) | 1.09 (0.89-1.32) |  | 101 | 0.82 (0.63-1.06) | 0.88 (0.67-1.15) | |  | 73 | 1.24 (0.88-1.75) | 1.39 (0.99-1.98)* | | |
| T2 (n=532) |  | 207 | | 1.00 (Referent) | 1.00 (Referent) |  | 121 | 1.00 (Referent) | 1.00 (Referent) | |  | 58 | 1.00 (Referent) | 1.00 (Referent) | | |
| T3 (n=626) |  | 249 | | 0.96 (0.80-1.15) | 0.97 (0.80-1.17) |  | 156 | 1.03 (0.81-1.30) | 1.01 (0.80-1.29) | |  | 61 | 0.83 (0.58-1.19) | 0.86 (0.60-1.24) | | |
| Per SD (3.8) |  | 669 | | 0.97 (0.90-1.04) | 0.92 (0.85-1.00)* |  | 378 | 1.09 (0.99-1.21) | 1.04 (0.93-1.15) | |  | 192 | 0.83 (0.72-0.95)** | 0.77 (0.66-0.89)*** | | |
| Alternative Healthy Eating Index – 2010 ^b^ | | | | | | | | | | | | | | | | |
| T1 (n=564) |  | 212 | | 0.98 (0.81-1.19) | 1.05 (0.87-1.27) |  | 115 | 0.99 (0.76-1.27) | 1.02 (0.78-1.32) | |  | 69 | 1.17 (0.83-1.65) | 1.42 (1.00-2.03)* | | |
| T2 (n=560) |  | 223 | | 1.00 (Referent) | 1.00 (Referent) |  | 121 | 1.00 (Referent) | 1.00 (Referent) | |  | 61 | 1.00 (Referent) | 1.00 (Referent) | | |
| T3 (n=589) |  | 234 | | 0.93 (0.77-1.11) | 0.95 (0.79-1.14) |  | 142 | 1.04 (0.82-1.33) | 1.04 (0.81-1.33) | |  | 62 | 0.89 (0.62-1.27) | 0.97 (0.67-1.39) | | |
| Per SD (9.7) |  | 669 | | 0.97 (0.90-1.05) | 0.94 (0.87-1.02) |  | 378 | 1.03 (0.93-1.14) | 0.99 (0.90-1.11) | |  | 192 | 0.87 (0.75-0.99)* | 0.80 (0.68-0.93)** | | |

*HR* hazard ratio-*CI* confidence intervals T1 decrease, T2 relatively no change, T3 increase

^a^ Adjusted for age-smoking habits-social class-physical activity-total energy intake and usual alcohol consumption, initial diet score

^b^ No adjustment for usual alcohol consumption in the multivariable model.

* p<0.05; ** p < 0.01; ***P < 0.001

**Online Resource 3** Cross-sectional relationship between diet scores (mean phase 2 and phase 3), and cardiovascular risk markers (mean phase 2 and phase 3) in the Caerphilly Prospective Study.

|  | Healthy Diet Indicator  (score range 0-6) | | | | | | | | | | | | | | | | | | | Dietary Approaches to Stop Hypertension score  (score range 11-35) | | | | | | | | | | | | | | | | | | | | | | Alternative Healthy Eating Index-2010 ^a^  (score range 10-80) | | | | | | | | | | | | | |
| --- | --- | --- | --- | --- | --- | --- | --- | --- | --- | --- | --- | --- | --- | --- | --- | --- | --- | --- | --- | --- | --- | --- | --- | --- | --- | --- | --- | --- | --- | --- | --- | --- | --- | --- | --- | --- | --- | --- | --- | --- | --- | --- | --- | --- | --- | --- | --- | --- | --- | --- | --- | --- | --- | --- | --- |
|  | T1  (n = 854) | T2  (n = 619) | | | | | | T3  (n = 394) | | | | | | Per sd  (1.0) | | | | | | T1  (n = 550) | | | | | | T2  (n = 604) | | | | | | T3  (n = 713) | | | | | Per sd (4.4) | | | | | T1  (n = 610 ) | | | | | | | | T2  (n = 624) | | | T3  (n = 633) | | Per sd (11.0) |
| Body Mass Index (kg/m^2^) | | | | | | | | | | | | | | | | | | | | | | | | | | | | | | | | | | | | | | | | | | | | | | | | | | | | | | | |
| Crude | Referent | 0.15  (-0.22, 0.52) | | | | | | 0.28  (-0.15, 0.71) | | | | | | | 0.04  (-0.12, 0.20) | | | | | Referent | | | | | | 1.04 **  (0.63, 1.45) | | | | | | 1.59 *  (1.19, 1.97) | | | | | 0.60 *  (0.44, 0.76) | | | | | Referent | | | | | 0.83**  (0.44, 1.23) | | | | | | 1.31 **  (0.92, 1.71) | | 0.50 **  (0.34, 0.67) |
| Adjusted ^b^ | Referent | -0.05  (-0.41, 0.31) | | | | | | -0.17  (-0.59, 0.25) | | | | | | | -0.12  (-0.28, 0.04) | | | | | Referent | | | | | | 0.65 **  (0.24, 1.06) | | | | | | 0.99 *  (0.56, 1.43) | | | | | 0.34*  (0.16, 0.52) | | | | | Referent | | | | | 0.46 **  (0.06, 0.87) | | | | | | 0.54 **  (0.09, 0.99) | | 0.15  (-0.04, 0.34) |
| Systolic blood pressure (mmHg) | | | | | | | | | | | | | | | | | | | | | | | | | | | | | | | | | | | | | | | | | | | | | | | | | | | | | | | |
| Crude | Referent | 0.04  (-2.02, 2.10) | | | | | | 0.48  (-1.89, 2.86) | | | | | | 0.07  (-0.83, 0.97) | | | | | | Referent | | | | | | 0.60  (-1.70, 2.89) | | | | | | -0.89  (-3.10, 1.32) | | | | | -0.25  (-1.15, 0.65) | | | | | Referent | | | | | 0.05  (-2.17, 2.27) | | | | | | -1.03  (-3.24, 1.18) | | -0.44  (-1.34, 0.46) |
| Adjusted | Referent | 0.69  (-1.30, 2.68) | | | | | | 1.27  (-1.06, 3.60) | | | | | | 0.40  (-0.49, 1.29) | | | | | | Referent | | | | | | -0.32  (-2.62, 1.97) | | | | | | -1.35  (-3.78, 1.09) | | | | | -0.26  (-1.27, 0.75) | | | | | Referent | | | | | -0.42  (-2.65, 1.82) | | | | | | -1.66  (-4.15, 0.83) | | -0.82  (-1.87, 2.24) |
| Diastolic blood pressure (mmHg) | | | | | | | | | | | | | | | | | | | | | | | | | | | | | | | | | | | | | | | | | | | | | | | | | | | | | | | |
| Crude | Referent | | 0.75  (-0.32, 1.81) | | | | | | 0.37  (-0.86, 1.60) | | | | | | 0.09  (-0.37, 0.56) | | | | | | Referent | | | | | | 0.97  (-0.23, 2.16) | | | | | 0.55  (-0.60, 1.70) | | | | | 0.07  (-0.40, 0.54) | | | | | Referent | | | | | 0.50  (-0.66 ,1.65) | | | | -0.31 **  (-1.46, 0.84) | | | | -0.15  (-0.62, 0.32) |
| Adjusted | Referent | | 0.74  (-0.30, 1.77) | | | | | | 0.16  (-1.05, 1.38) | | | | | | 0.07  (-0.39, 0.54) | | | | | | Referent | | | | | | 0.06  (-1.14, 1.26) | | | | | -0.87  (-2.14, 0.40) | | | | | -0.47  (-0.99, 0.06) | | | | | Referent | | | | | -0.28  (-1.44, 0.88) | | | | -1.47  (-2.77, -0.18) | | | | -0.66 **  (-1.21, -0.12) |
| Total cholesterol (mmol/L) | | | | | | | | | | | | | | | | | | | | | | | | | | | | | | | | | | | | | | | | | | | | | | | | | | | | | | | |
| Crude | Referent | | | 0.03  (-0.07, 0.14) | | | | | | -0.06  (-0.18, 0.06) | | | | | | -0.03  (-0.08, 0.02) | | | | | | Referent | | | | | | -0.06  (-0.18, 0.06) | | | | | 0.01  (-0.10, 0.12) | | | | | 0.02  (-0.03, 0.07) | | | | | Referent | | | | -0.08  (-0.19, 0.03) | | | | 0.01  (-0.10, 0.12) | | | | -0.01  (-0.05, 0.04) |
| Adjusted | Referent | | | 0.03  (-0.08, 0.13) | | | | | | -0.06  (-0.19, 0.06) | | | | | | -0.03  (-0.08, 0.02) | | | | | | Referent | | | | | | -0.06  (-0.19, 0.06) | | | | | -0.01  (-0.14, 0.12) | | | | | 0.02  (-0.04, 0.07) | | | | | Referent | | | | -0.10  (-0.22, 0.02) | | | | -0.01  (-0.14, 0.12) | | | | -0.02  (-0.08, 0.04) |
| LDL- cholesterol (mmol/L) | | | | | | | | | | | | | | | | | | | | | | | | | | | | | | | | | | | | | | | | | | | | | | | | | | | | | | | |
| Crude | Referent | | | | 0.05  (-0.05, 0.14) | | | | | | -0.03  (-0.14, 0.08) | | | | | | -0.02  (-0.06, 0.02) | | | | | | Referent | | | | | | -0.01  (-0.12, 0.09) | | | | | 0.03  (-0.07, 0.14) | | | | | 0.03  (-0.01, 0.07) | | | | | Referent | | | | 0.04  (-0.07, 0.14) | | | | 0.09  (-0.01, 0.20) | | | 0.04  (-0.01, 0.08) |
| Adjusted | Referent | | | | 0.04  (-0.06, 0.13) | | | | | | -0.05  (-0.16, 0.07) | | | | | | -0.03  (-0.07, 0.02) | | | | | | Referent | | | | | | -0.05  (-0.16, 0.06) | | | | | -0.04  (-0.16, 0.08) | | | | | 0.01  (-0.04, 0.06) | | | | | Referent | | | | 0.01  (-0.10, 0.12) | | | | 0.04  (-0.08, 0.17) | | | 0.02  (-0.03, 0.07) |
| HDL-cholesterol (mmol/L) | | | | | | | | | | | | | | | | | | | | | | | | | | | | | | | | | | | | | | | | | | | | | | | | | | | | | | | |
| Crude | Referent | | | | -0.01  (-0.04, 0.01) | | | | | | -0.02  (-0.05, 0.01) | | | | | | -0.01  (-0.02, 0.01) | | | | | | Referent | | | | | | 0.01  (-0.02, 0.04) | | | | | -0.02  (-0.05, 0.01) | | | | | -0.004  (-0.02, 0.001) | | | | | Referent | | | | -0.02  (-0.05, 0.004) | | | | -0.05*  (-0.08, -0.02) | | | -0.02 *  (-0.03, -0.01) |
| Adjusted | Referent | | | | -0.01  (-0.03, 0.02) | | | | | | -0.02  (-0.04, 0.01) | | | | | | -0.01  (-0.02, 0.004) | | | | | | Referent | | | | | | 0.02  (-0.01, 0.05) | | | | | 0.001  (-0.03, 0.03) | | | | | 0.003  (-0.01, 0.02) | | | | | Referent | | | | -0.02  (-0.05, 0.01) | | | | -0.04 **  (-0.07, -0.01) | | | -0.02 *  (-0.04, -0.01) |
| Log triacylglycerol (mmol/L) | | | | | | | | | | | | | | | | | | | | | | | | | | | | | | | | | | | | | | | | | | | | | | | | | | | | | | | |
| Crude | Referent | | | | 0.01  (-0.04, 0.06) | | | | | | -0.02  (-0.08, 0.04) | | | | | | -0.01  (-0.03, 0.02) | | | | | | Referent | | | | | | -0.02  (-0.07, 0.04) | | | | | -0.03  (-0.08, 0.02) | | | | | -0.01  (-0.04, 0.01) | | | | | Referent | | | | -0.09  (-0.14, -0.04)* | | | | | | -0.08*  (-0.13, -0.02) | -0.03*  (-0.05, -0.01) |
| Adjusted | Referent | | | | 0.02  (-0.03, 0.06) | | | | | | -0.00  (-0.06, 0.06) | | | | | | 0.00  (-0.02, 0.02) | | | | | | Referent | | | | | | -0.03  (-0.09, 0.03) | | | | | -0.04  (-0.10, 0.02) | | | | | -0.02  (-0.04, 0.01) | | | | | Referent | | | | -0.12 *  (-0.17, -0.07) | | | | | | -0.11*  (-0.16, -0.05) | -0.04 *  (-0.07, -0.02) |
| Blood glucose (mmol/L) | | | | | | | | | | | | | | | | | | | | | | | | | | | | | | | | | | | | | | | | | | | | | | | | | | | | | | | |
| Crude | Referent | | | | | -0.04  (-0.12, 0.04) | | | | | | -0.04  (-0.13, 0.05) | | | | | | -0.02  (-0.05, 0.02) | | | | | | Referent | | | | | | 0.05  (-0.04, 0.14) | | | | | 0.001  (-0.09, 0.09) | | | | | -0.004  (-0.04, 0.03) | | | | | Referent | | | 0.04  (-0.05, 0.13) | | | | | | 0.01  (-0.08, 0.10) | 0.01  (-0.02, 0.05) |
| Adjusted | Referent | | | | | -0.04  (-0.12, 0.04) | | | | | | -0.07  (-0.16, 0.02) | | | | | | -0.03  (-0.06, 0.01) | | | | | | Referent | | | | | | 0.004  (-0.09, 0.09) | | | | | -0.08  (-0.18, 0.01) | | | | | -0.04**  (-0.08, -0.01) | | | | | Referent | | | -0.02  (-0.11, 0.06) | | | | | | -0.06  (-0.16, 0.03) | -0.02  (-0.06, 0.02) |
| Log C – Reactive Protein (mg/L) | | | | | | | | | | | | | | | | | | | | | | | | | | | | | | | | | | | | | | | | | | | | | | | | | | | | | | | |
| Crude | Referent | | | | | | -0.02  (-0.15, 0.11) | | | | | | -0.27 *  (-0.41, -0.12) | | | | | | -0.11 *  (-0.16, -0.05) | | | | | | Referent | | | | | | -0.10  (-0.24, 0.04) | | | | | -0.36 *  (-0.50, -0.23) | | | | | -0.17 *  (-0.22, - 0.11) | | | | | Referent | | | -0.17 **  (-0.31, 0.03) | | | | | -0.28 *  (-0.41, -0.14) | -0.13*  (-0.19, -0.08) |
| Adjusted | Referent | | | | | | 0.02  (-0.10, 0.14) | | | | | | -0.18 *  (-0.32, -0.04) | | | | | | -0.07*  (-0.13, -0.02) | | | | | | Referent | | | | | | -0.06  (-0.20, 0.08) | | | | | -0.27 *  (-0.42, -0.13) | | | | | -0.13*  (-0.19, -0.07) | | | | | Referent | | | -0.14 **  (-0.28, 0.01) | | | | | -0.20 *  (-0.34, -0.05) | -0.10 *  (-0.16, -0.04) |

Crude and adjusted change in cardiovascular risk markers, expressed using the regression coefficients (95 % CI). Adjusted for age, smoking status, social class, physical activity, total energy intake, usual alcohol consumption and BMI.

* *P* < 0.01 and *P* trend < 0.01; ** *P* < 0.05

^a^ No adjustment for usual alcohol consumption in the multivariable model.

**Online Resource 4** Hazard ratios with 95 % CIs for cardiovascular disease, coronary heart disease and stroke by diet scores in 586 middle-aged men who had no indication of preclinical cardiovascular disease at Phase 2 and Phase 3 in the Caerphilly Prospective Study.

|  |  | Cardiovascular disease | | | |  | Coronary heart disease | | | |  | Stroke | | | | |
| --- | --- | --- | --- | --- | --- | --- | --- | --- | --- | --- | --- | --- | --- | --- | --- | --- |
|  |  | Events | Crude HR  (95 % CI) | | Adjusted HR^a^ (95 % CI) |  | Events | Crude HR (95 % CI) | Adjusted HR^a^ (95 % CI) | |  | Events | Crude HR  (95 % CI) | | | Adjusted HR^a^ (95 % CI) |
| Healthy Diet Indicator | | | | | | | | | | | | | | | | |
| T1 (n=257) |  | 85 | 1.00 (Referent) | | 1.00 (Referent) |  | 41 | 1.00 (Referent) | | 1.00 (Referent) |  | 31 | 1.00 (Referent) | | 1.00 (Referent) | |
| T2 (n=201) |  | 59 | 0.78 (0.55-1.08) | | 0.88 (0.63-1.24) |  | 29 | 0.79 (0.49-1.27) | | 0.84 (0.51-1.37) |  | 17 | 0.62 (0.34-1.11) | | 0.75 (0.41-1.37) | |
| T3 (n=128) |  | 31 | 0.70 (0.47-1.06) | | 0.83 (0.54-1.27) |  | 12 | 0.58 (0.31-1.11) | | 0.66 (0.34-1.29) |  | 17 | 1.02 (0.57-1.86) | | 1.26 (0.67-2.35) | |
| Per SD (1.0) |  | 175 | 0.88 (0.76-1.04) | | 0.93 (0.80-1.09) |  | 82 | 0.85 (0.67-1.07) | | 0.88 (0.69-1.11) |  | 65 | 0.98 (0.77-1.25) | | 1.04 (0.81-1.33) | |
| Dietary Approaches to Stop Hypertension score | | | | | | | | | | | | | | | | |
| T1 (n=169) |  | 56 | 1.00 (Referent) | 1.00 (Referent) | |  | 24 | 1.00 (Referent) | 1.00 (Referent) | |  | 20 | 1.00 (Referent) | 1.00 (Referent) | | |
| T2 (n=180) |  | 52 | 0.75 (0.52-1.10) | 0.79 (0.53-1.18) | |  | 23 | 0.78 (0.44-1.38) | 0.85 (0.46-1.55) | |  | 20 | 0.81 (0.43-1.50) | 0.75 (0.39-1.44) | | |
| T3 (n=237) |  | 67 | 0.64 (0.45-0.92) | 0.64 (0.42-0.96) | |  | 35 | 0.78 (0.46-1.31) | 0.81 (0.43-1.50) | |  | 25 | 0.67 (0.37-1.21) | 0.62 (0.31-1.21) | | |
| Per SD (4.4) |  | 175 | 0.82 (0.71-0.95) | 0.82 (0.69-0.97) | |  | 82 | 0.91 (0.74-1.13) | 0.93 (0.72-1.20) | |  | 65 | 0.80 (0.63-1.02) | 0.76 (0.59-1.00) | | |
| Alternative Healthy Eating Index – 2010 ^b^ | | | | | | | | | | | | | | | | |
| T1 (n=179) |  | 57 | 1.00 (Referent) | 1.00 (Referent) | |  | 25 | 1.00 (Referent) | 1.00 (Referent) | |  | 19 | 1.00 (Referent) | 1.00 (Referent) | | |
| T2 (n=198) |  | 61 | 0.83 (0.58-1.19) | 0.88 (0.60-1.31) | |  | 26 | 0.81 (0.47-1.40) | 0.82 (0.46-1.48) | |  | 30 | 1.21 (0.68-2.15) | 1.26 (0.67-2.39) | | |
| T3 (n=209) |  | 57 | 0.63 (0.43-0.90) | 0.63 (0.41-0.98) | |  | 31 | 0.78 (0.46-1.32) | 0.77 (0.41-1.47) | |  | 16 | 0.52 (0.27-1.02) | 0.52 (0.24-1.15) | | |
| Per SD (11.0) |  | 175 | 0.83 (0.72-0.96) | 0.82 (0.68-0.99) | |  | 82 | 0.91 (0.73-1.13) | 0.93 (0.71-1.22) | |  | 65 | 0.79 (0.62-1.01) | 0.74 (0.55-1.00) | | |

*HR* hazard ratio-*CI* confidence intervals

^a^ Adjusted for age-smoking habits-social class-physical activity-total energy intake and usual alcohol consumption.

^b^ No adjustment for usual alcohol consumption in the multivariable model.

**Online Resource 5** Hazard ratios with 95 % CIs for cardiovascular disease, coronary heart disease and stroke by diet scores in 1,268 middle-aged men who had have an indication of preclinical cardiovascular disease at Phase 2 and Phase 3 in the Caerphilly Prospective Study.

|  |  | Cardiovascular disease | | | |  | Coronary heart disease | | | |  | Stroke | | | | |
| --- | --- | --- | --- | --- | --- | --- | --- | --- | --- | --- | --- | --- | --- | --- | --- | --- |
|  |  | Events | Crude HR  (95 % CI) | | Adjusted HR^a^ (95 % CI) |  | Events | Crude HR (95 % CI) | Adjusted HR^a^ (95 % CI) | |  | Events | Crude HR  (95 % CI) | | | Adjusted HR^a^ (95 % CI) |
| Healthy Diet Indicator | | | | | | | | | | | | | | | | |
| T1 (n=591) |  | 257 | 1.00 (Referent) | | 1.00 (Referent) |  | 151 | 1.00 (Referent) | | 1.00 (Referent) |  | 74 | 1.00 (Referent) | | 1.00 (Referent) | |
| T2 (n=412) |  | 181 | 1.02 (0.84-1.24) | | 1.05 (0.87-1.28) |  | 108 | 1.04 (0.81-1.33) | | 1.06 (0.82-1.36) |  | 49 | 0.97 (0.68-1.39) | | 1.03 (0.71-1.49) | |
| T3 (n=265) |  | 107 | 0.81 (0.65-1.02) | | 0.91 (0.72-1.15) |  | 62 | 0.81 (0.60-1.09) | | 0.91 (0.67-1.23) |  | 20 | 0.52 (0.32-0.86) | | 0.58 (0.35-0.97) | |
| Per SD (1.0) |  | 545 | 0.97 (0.89-1.05) | | 1.00 (0.92-1.09) |  | 321 | 0.96 (0.86-1.06) | | 0.99 (0.89-1.11) |  | 143 | 0.89 (0.76-1.05) | | 0.93 (0.78-1.10) | |
| Dietary Approaches to Stop Hypertension score | | | | | | | | | | | | | | | | |
| T1 (n=377) |  | 170 | 1.00 (Referent) | 1.00 (Referent) | |  | 96 | 1.00 (Referent) | 1.00 (Referent) | |  | 56 | 1.00 (Referent) | 1.00 (Referent) | | |
| T2 (n=420) |  | 175 | 0.81 (0.65-1.00) | 0.85 (0.68-1.06) | |  | 114 | 0.93 (0.71-1.23) | 0.95 (0.72-1.27) | |  | 40 | 0.56 (0.38-0.85) | 0.61 (0.40-0.94) | | |
| T3 (n=471) |  | 200 | 0.72 (0.58-0.88) | 0.86 (0.68-1.09) | |  | 111 | 0.71 (0.54-0.94) | 0.84 (0.62-1.15) | |  | 47 | 0.50 (0.34-0.74) | 0.58 (0.37-0.90) | | |
| Per SD (4.4) |  | 545 | 0.88 (0.80-0.96) | 0.96 (0.87-1.07) | |  | 321 | 0.85 (0.76-0.96) | 0.92 (0.81-1.05) | |  | 143 | 0.81 (0.68-0.95) | 0.89 (0.73-1.08) | | |
| Alternative Healthy Eating Index – 2010 ^b^ | | | | | | | | | | | | | | | | |
| T1 (n=386) |  | 165 | 1.00 (Referent) | 1.00 (Referent) | |  | 100 | 1.00 (Referent) | 1.00 (Referent) | |  | 50 | 1.00 (Referent) | 1.00 (Referent) | | |
| T2 (n=459) |  | 199 | 0.90 (0.74-1.11) | 0.91 (0.73-1.13) | |  | 114 | 0.85 (0.65-1.12) | 0.83 (0.63-1.09) | |  | 52 | 0.78 (0.53-1.16) | 0.82 (0.55-1.23) | | |
| T3 (n=423) |  | 181 | 0.79 (0.64-0.98) | 0.86 (0.68-1.10) | |  | 107 | 0.78 (0.60-1.03) | 0.81 (0.59-1.11) | |  | 41 | 0.58 (0.38-0.88) | 0.66 (0.41-1.06) | | |
| Per SD (11.0) |  | 545 | 0.92 (0.84-1.00) | 0.97 (0.87-1.08) | |  | 321 | 0.92 (0.82-1.03) | 0.96 (0.83-1.09) | |  | 143 | 0.79 (0.66-0.93) | 0.83 (0.68-1.02) | | |

*HR* hazard ratio-*CI* confidence intervals

^a^ Adjusted for age-smoking habits-social class-physical activity-total energy intake and usual alcohol consumption.

^b^ No adjustment for usual alcohol consumption in the multivariable model.

Online Resource 6 Sensitivity analyses on the DASH score; Hazard ratios with 95 % CIs for cardiovascular disease, coronary heart disease and stroke by excluding the milk component of the DASH score in the Caerphilly Prospective Study.

|  | Tertiles of DASH score | | | | | continuous  Per SD (4.4) |
| --- | --- | --- | --- | --- | --- | --- |
|  | T1 (n=550) | T2 (n=604) | | T3 (n=713) | |  |
| Cardiovascular disease | | | | | | |
| Events | 227 | 228 | | 270 | | 725 |
| HR^a^ DASH score | 1.00 (Referent) | 0.85 (0.70-1.03) | | 0.81 (0.66-0.99) | | 0.92 (0.84-1.00) |
| Excluding milk | 1.00 (Referent) | 0.88 (0.72-1.06) | | 0.78 (0.63-0.97) | | 0.91 (0.84-0.99) |
| Coronary heart disease | | | | | | |
| Events | 121 | 138 | | 148 | | 407 |
| HR^a^ DASH score | 1.00 (Referent) | 0.97 (0.75-1.25) | | 0.84 (0.64-1.10) | | 0.91 (0.81-1.02) |
| Excluding milk | 1.00 (Referent) | 0.99 (0.77-1.28) | | 0.83 (0.62-1.12) | | 0.90 (0.80-1.01) |
| Stroke | | | | | | |
| Events | 76 | | 60 | | 73 | 209 |
| HR^a^ DASH score | 1.00 (Referent) | | 0.66 (0.46-0.94) | | 0.61 (0.42-0.88) | 0.85 (0.73-1.00) |
| Excluding milk | 1.00 (Referent) | | 0.64 (0.45-0.90) | | 0.53 (0.35-0.79) | 0.85 (0.72-1.00) |

*HR* hazard ratio, *CI* confidence intervals

^a^ Adjusted for age, smoking habits, social class, physical activity, total energy intake and usual alcohol consumption.
